# A bizarre Eocene dasyatoid batomorph (Elasmobranchii, Myliobatiformes) from the Bolca Lagerstätte (Italy) reveals a new, extinct body plan for stingrays

**DOI:** 10.1038/s41598-019-50544-y

**Published:** 2019-10-01

**Authors:** Giuseppe Marramà, Giorgio Carnevale, Luca Giusberti, Gavin J. P. Naylor, Jürgen Kriwet

**Affiliations:** 1 University of Vienna, Department of Palaeontology, Vienna, 1090 Austria; 2 Università degli Studi di Torino, Dipartimento di Scienze della Terra, Torino, 10125 Italy; 3 Università degli Studi di Padova, Dipartimento di Geoscienze, Padova, 35131 Italy; 4 University of Florida, Florida Museum of Natural History, Gainesville, 32611 Florida USA

**Keywords:** Ichthyology, Palaeontology

## Abstract

In the last few years, the detailed revision of the Eocene cartilaginous fishes (Chondrichthyes) from the Bolca Lagerstätte (Italy) has provided new insights into the fish biodiversity of the western Tethys. The morphological analysis of three previously undescribed specimens from the Pesciara deposit of Bolca revealed the existence of a new stingray taxon, †*Lessiniabatis aenigmatica* gen. et sp. nov., which is unique among the myliobatiform batoids in having the following unique combination of characters: low number of vertebrae posterior to the pelvic girdle (65–68); thoracolumbar synarcual extending backward beyond the pelvic girdle; tail extremely short not protruding from the posterior edge of the pectoral disc; radials proximally fused to each other; pelvic girdle extremely small and strongly arched; dorsal and caudal fins absent; tail stings and cartilaginous tail rod absent; and teeth of dasyatoid morphology with smooth enameloid surface. The phylogenetic analysis suggests that †*Lessiniabatis* gen. nov. is deeply nested within the benthic stingrays (Dasyatoidea) representing the sister to all dasyatids and potamotrygonids. Its unique anatomy clearly reveals the existence of a new hitherto unknown body plan experimented by benthic stingrays, whose evolution can be possibly linked to the adaptive fish radiation in the aftermath of the end-Cretaceous extinction.

## Introduction

Stingrays (order Myliobatiformes) are a speciose group of batoid fishes comprising more than 360 extant species arranged in 11 families^1,2^. Although their interrelationships are still ambiguous with contrasting results emerging from molecular and morphological studies^1,3–6^, stingrays can be unambiguously regarded as a monophyletic clade of batoids characterized by a series of derived features, including the presence of one or more serrated tail stings, a second (thoracolumbar) synarcual, absence of rostral cartilage, and other unique features of the gill arches and pectoral girdle^3,4,7–10^. Although the fossil record of stingrays is abundant and can be traced back to the Lower Cretaceous^11^, it is strongly biased toward isolated teeth with the exception of a few Cenozoic records represented by complete articulated skeletons^3,6,12–15^. Myliobatiforms may have experienced a first radiation on the latest Cretaceous with the appearance and diversification of pelagic/benthopelagic durophagous taxa, followed by a second wave of radiation during the early Paleogene with the appearance and radiation of the planktivorous stingrays^5,16,17^. Their early Paleogene radiation co-occurred with many other remarkable diversifications of bony and cartilaginous fish lineages driven by the availability of new ecological niches associated with the establishment of modern coral reefs and the removal by extinction of many Cretaceous clades^18–24^. The celebrated Eocene (Ypresian, ca. 49 Ma^25,26^) Konservat‐Lagerstätte of Pesciara of Bolca in northeastern Italy is known for the outstanding diversity and preservational quality of bony and cartilaginous fishes, which provide evidence of the recovery of shallow marine settings associated with reefs after the K/Pg boundary^27^. The Bolca fish assemblage also documents the emergence of peculiar body plans and evolutionary innovations, which are indicative of a considerable experimentation of new ecological strategies. Examples include stem flatfishes (Pleuronectiformes) showing gradual evolution of asymmetry^28,29^, curious ribbon-like morphology of †*Bajaichthys*, very different from the deep bodies of modern zeiforms^30^, basal members of the hyperdiverse benthic gobioids^31^, a variety of bizarre anglerfishes^32–35^ and plectognaths^36^, or the peculiar paralepidid aulopiform †*Holosteus* whose body plan mimics that of pike-like ambush predators^23^. In this study, another one in a series of papers highlighting the palaeobiodiversity of the Eocene batoids from the Bolca Lagerstätte^6,13,15,24^, we describe in detail the anatomy and the phylogenetic relationships of a new stingray genus whose unique body plan, unknown in other extinct or living myliobatiform taxa, supports the idea that evolutionary novelties and innovations also occurred in cartilaginous fishes in the aftermath of the end-Cretaceous extinction, leading to the exploitation of new ecological strategies.

## Results

### Systematic palaeontology

Class Chondrichthyes Huxley, 1880.

Superorder Batomorphii Cappetta, 1980.

Order Myliobatiformes Compagno, 1973.

Superfamily Dasyatoidea Compagno, 1973.

Family incertae sedis.

Genus †*Lessiniabatis* gen. nov.

urn:lsid:zoobank.org:pub:72B8B7EE-0D87-4864-B31A-5695AAEE89C0.

*Type species. †Lessiniabatis aenigmatica sp. nov*.

#### Etymology

After Lessinia, the Italian geographic area where the Bolca Lagerstätte is located, and from the Ancient Greek word ‘βατίς’ (*batis*), meaning ray or skate; gender feminine.

#### Diagnosis

A dasyatoid stingray unique in having a thoracolumbar synarcual extending backward beyond the pelvic girdle, a tail that is extremely short and not protruding from the posterior edge of the pectoral disc, and pectoral radials that are proximally fused with one another. Moreover, †*Lessiniabatis* gen. nov. is characterized by the following combination of traits: low number of vertebrae posterior to the pelvic girdle (65–68); dorsal and caudal fins absent; pelvic girdle extremely small and strongly arched; tail stings and cartilaginous tail rod absent; teeth with dasyatoid morphology; enameloid surface smooth; lingual tooth surface broad and slightly convex; labial tooth surface slightly concave; labial and lingual tooth faces not steep in lateral profile; tooth root bilobed, with a central foramen; holaulacorhizid root type with an elongated pulp cavity; sparse, star-shaped dermal denticles covering the whole body; about 130–135 pectoral-fin radials (of which 59–61 are propterygial, 16–18 are mesopterygial, and 54–57 are metapterygial); about 120 vertebral centra.

#### Composition of the genus

The genus is represented only by the type species †*Lessianabatis aenigmatica* sp. nov.

†*Lessiniabatis aenigmatica* sp. nov.

(Figures 1–7)

*Urolophus crassicaudatus* (Blainville, 1818): Raux (2014), Figs 1, 7–9, 11^37^.

“*Urolophus*” *crassicaudatus* (Blainville, 1818): Marramà *et al*. (2018), Fig. 9A,B, non C-D^15^.

#### Etymology

After the Latin word ‘*aenigmaticus*’, *-a*, meaning enigmatic, puzzling, referring to its peculiar anatomy; gender feminine.

#### Holotype

**Figure 1:**
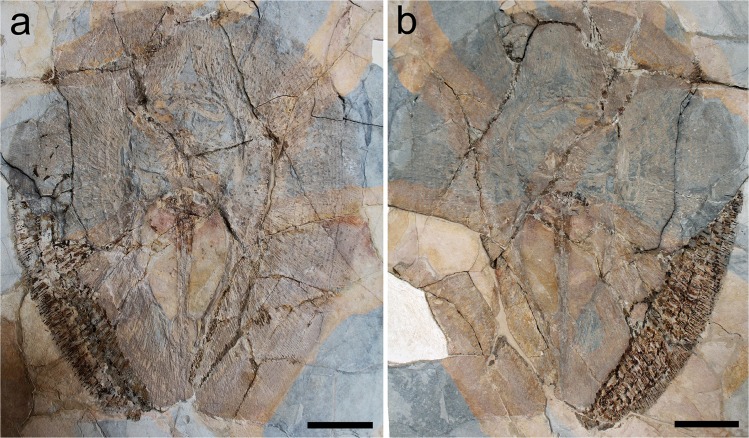
†*Lessiniabatis aenigmatica* gen. et sp. nov. from the Eocene of Bolca Lagerstätte. (**a,b**) the holotype MNHN F.Bol.566 in part and counterpart. Scale bars equal 100 mm.

MNHN F.Bol.566 (ex 11001 and 10997), nearly complete articulated skeleton, in part (566B) and counterpart (566 A); 466.8 mm DW (Fig. 1).

#### Paratypes

**Figure 2:**
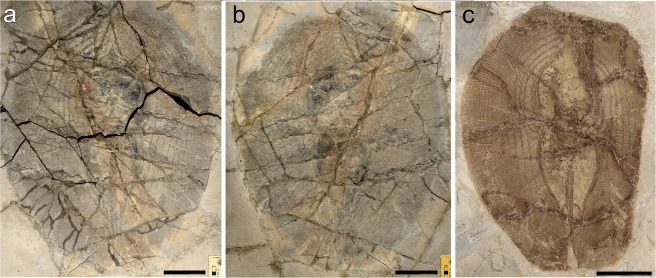
†*Lessiniabatis aenigmatica* gen. et sp. nov. from the Eocene of Bolca Lagerstätte. (**a,b**) the paratype MSNFI IGF 103555 in part and counterpart; (**c**) the paratype MFSN GP.864. Scale bars equal 100 mm.

MSNFI IGF 103555, nearly complete articulated skeleton in part and counterpart, 568.2 mm DW (Fig. 2a,b); MFSN GP.864, partially articulated skeleton on a single slab, with most of the neurocranium and pectoral girdle being disarticulated; 375.1 mm DW (Fig. 2c).

#### Type locality and horizon

Pesciara site, Bolca Konservat-Lagerstätte, Italy; lower Eocene, upper Ypresian, SB 11 and NP 14a Zones^25^.

#### Diagnosis

Same as genus (monotypic genus).

#### Remarks

The holotype we designate herein for †*Lessiniabatis aenigmatica* gen. et sp. nov., MNHN F.Bol.566 (previously labelled as 10997–11001), is the specimen indicated as the holotype of †*Urolophus crassicaudatus* (Blainville, 1818)^38^ in the collections of the Museum National d’Histoire Naturelle of Paris (https://science.mnhn.fr/taxon/species/urolophus/crassicaudatus). However, as already suggested by Eastman (1904, p. 25; 1905, p. 9)^39,40^, this specimen cannot be identified as the type of †*Urolophus crassicaudatus*. In fact, the original anatomical description and measurements of the specimen given by Blainville (1818, p. 337)^38^ (originally described as †*Trygonobatus crassicaudatus*) clearly do not fit with those of MNHN F.Bol.566. The author shortly described, without any illustration, a fossil ray having a thick and robust tail (whence *crassicaudatus*) with trace of a tail sting, with a body 15 inches long (about 38 cm) and 16 broad (about 41 cm)^38^. Conversely, MNHN F.Bol.566 shows a very short and slender tail (without any trace of tail sting) and the body is longer (about 55 cm) than broad (about 47 cm). As the specimen originally described by Blainville^38^ clearly does not correspond to MNHN F.Bol.566, as well as none of the other rays from Bolca housed at the MNHN we examined, the original holotype of *T. crassicaudatus* is to be considered lost. A detailed nomenclatural and taxonomical revision of this taxon as later described and figured by Heckel^41,42^ and Jaekel^43^ is currently underway.

## Description

†*Lessiniabatis aenigmatica* gen. et sp. nov. is represented by three nearly complete and articulated specimens. The holotype (MNHN F.Bol.566) is the most complete, preserving most of the anatomical features (Fig. 1). The paratypes (MSNFI IGF 103555 and MFSN GP.864) are less complete (Fig. 2). Although the outline and morphology of the pectoral disc are clearly recognizable, the neurocranium and part of the pectoral region of the specimen MFSN GP.864 underwent severe disarticulation possibly due to taphonomic processes. However, in combination, the three specimens allowed the recognition and description of several skeletal and dental characters, which are useful to distinguish and separate this new stingray from any other known living and fossil myliobatiform taxon. Detailed counts and measurements are provided in the Supplementary Information. The holotype MNHN F.Bol.566 measures about 55 cm in disc length and 47 cm in disc width; MSNFI IGF 103555 is about 74 and 57 cm in disc length and width, respectively; MFSN GP.864 is about 47 and 38 cm in disc length and width, respectively.

The pectoral disc of †*Lessiniabatis* gen. nov. is almost ovoid in outline, longer than wide (117–130% of DW) and reaching the maximum width at about mid-length. The tail is extremely short (25–34% of DW), and its distal tip rests within the pectoral disc. †*Lessiniabatis aenigmatica* gen. et sp. nov. seems to lack any dorsal and caudal fins or tail folds. The skeleton is highly calcified and most of the elements, including jaws, hyomandibulae, synarcuals, pectoral and pelvic girdles, show the typical prismatic calcification of elasmobranchs.

### Neurocranium

**Figure 3:**
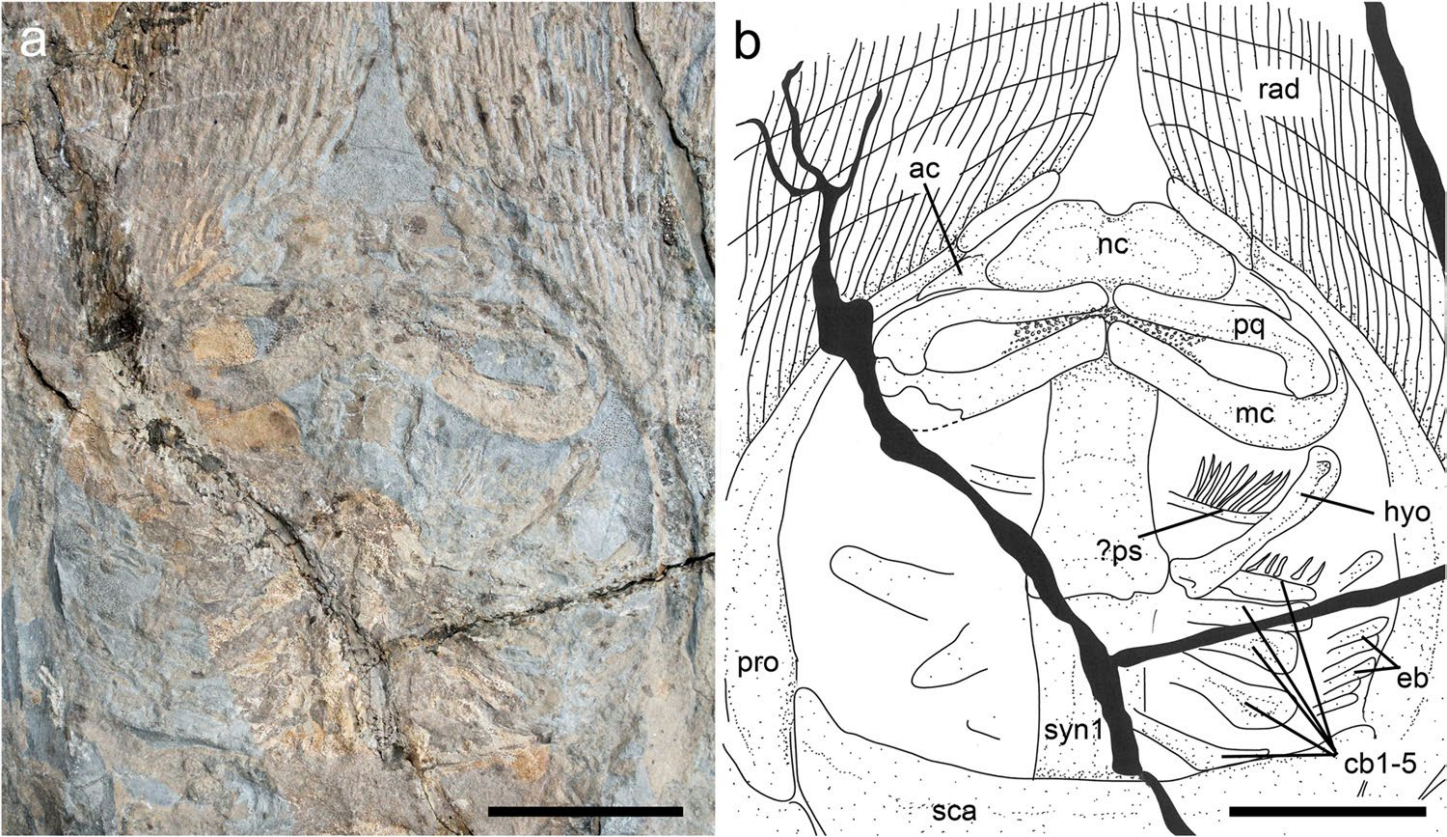
†*Lessiniabatis aenigmatica* gen. et sp. nov. from the Eocene of Bolca Lagerstätte. (**a**) Detail of head region and gill arches in MNHN F.Bol.566; (**b**) interpretative drawing. Abbreviations: ac, antorbital cartilage; cb, ceratobranchials; eb, epibranchials; hyo, hyomandibula; mc, Meckel’s cartilage; nc, nasal capsules; pq, palatoquadrate; pro, propterygium; rad, pectoral radials; sca, scapulocoracoid; syn1, cervicothoracic synarcual. Scale bars equal 50 mm.

The morphology of the neurocranium is recognizable in the holotype MNHN F.Bol.566 and the paratype MSNFI IGF 103555. It is antero-posteriorly elongate, longer than wide, with the greatest width at the level of the nasal capsules (Fig. 3). As in all stingrays^8,44^ the rostral cartilage is absent. The nasal capsules are transversely broad, ovoid in shape and their anterior margin is rounded and biconvex, with a wide anterior median indentation. The neurocranium lacks the typical anterior processes of some pelagic stingrays (*Rhinoptera* and *Mobula*). The preorbital, supraorbital and postorbital processes of the neurocranium are difficult to detect. The orbital region, in part obscured by the jaws, appears longer than wide. The outline of the otic capsules is difficult to recognize but they possibly provide articulation for the proximal portion of the hyomandibulae. The antorbital cartilages are broad, not branched and subtriangular in shape. Their maximum width is located at the level of the articulation with the nasal capsules. They taper distally and articulate with the propterygia through their outer margin.

### Jaws

The upper and lower jaws of †*Lessiniabatis* gen. nov. extend laterally and occupy almost the entire space between the propterygia (Fig. 3). Although the jaws are reinforced by prismatic calcification as in most of the stingrays, they are not massive like in potamotrygonids or dasyatids but quite slender and elongate. Near the symphysis, the lower jaws are slender, not thickened and unfused. The oral diastema width is greater or at least equal to the occlusal width. The palatoquadrate is labio-lingually compressed, narrower and smaller than the Meckel’s cartilage, with its dorsal flange being relatively convex. Small anterior processes of the Meckel’s cartilage of some dasyatoids like *Trygonoptera*, *Taeniura* and *Neotrygon*^17^ are absent in †*Lessiniabatis* gen. nov. At the same time, the medial symphyseal processes typical of *Dasyatis* and *Himantura*^17^, or the ventro-lateral processes of the mandibular cartilage of †*Asterotrygon*, *Taeniura*, potamotrygonids, and other non-myliobatid stingrays^3^ are difficult to discern in the available material. However, the lateral projections of the Meckel’s cartilage (or ‘wing-like processes’) are clearly absent in †*Lessiniabatis* gen. nov.

### Hyoid and gill arches

The hyomandibula appears slender, narrow at about its mid-length, slightly arched and with a concave inner margin (Fig. 3). The hyomandibula projects antero-laterally, almost reaching the mesial margin of the propterygium. A small gap is present between the distal end of the hyomandibula and the Meckel’s cartilage, suggesting that they were not directly articulated originally and that the hyomandibulae possibly articulated with the lower jaws through the strong, robust and non-mineralized hyomandibular-Meckelian ligament. However, the angular cartilages are clearly absent (conversely, these structures are easy to recognize if present in fossil stingrays^3^). Also the presence of the secondary hyomandibular cartilages characteristic of *Urolophus* and pelagic stingrays^3,9,45^ can be excluded. The ventral gill arches of †*Lessiniabatis* gen. nov. are poorly preserved and their morphology is difficult to recognize. There are possibly five pairs of ceratobranchials, but it is unclear whether the first one is fused to the pseudohyoid as in most of the dasyatoids, or if the last two ceratobranchials are ankylosed or fused to each other in their proximal portion. However, it is clear that the fifth ceratobranchial pair articulates with the anterior margin of the scapulocoracoid. Some epibranchials are associated with ceratobranchials. Filamentous branchial rays associated with the pseudohyoid and ceratobranchials can be also recognized.

### Synarcuals and vertebral column

**Figure 4:**
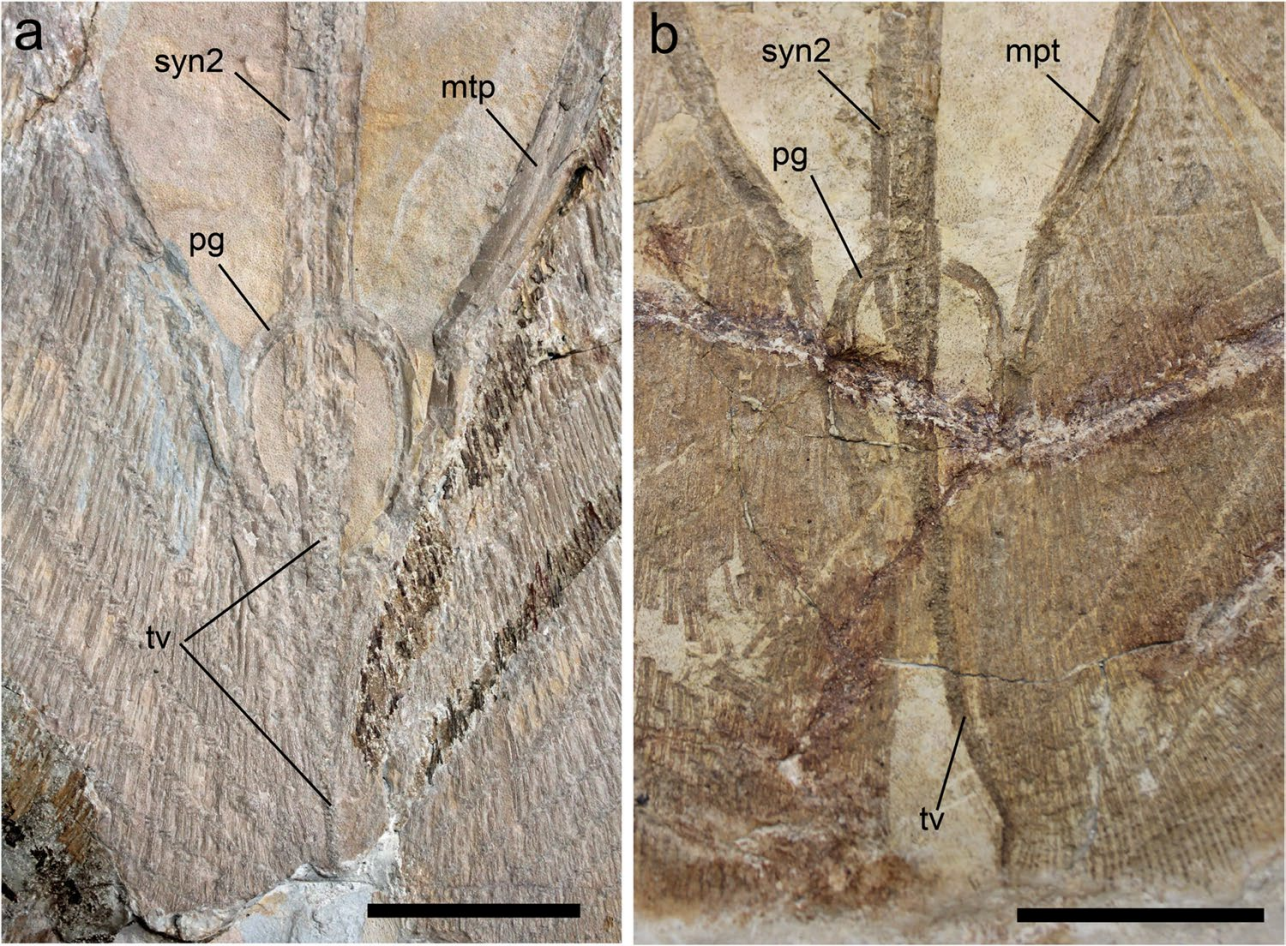
†*Lessiniabatis aenigmatica* gen. et sp. nov. from the Eocene of Bolca Lagerstätte. Details of the pelvic girdle and tail region in MNHN F.Bol.566 (**a**) and MFSN GP.864 (**b**). Abbreviations: mpt, metapterygium; pg, pelvic girdle; syn2, thoracolumbar synarcual; tv, tail vertebrae. Scale bars equal 50 mm.

The morphology of the anterior (cervicothoracic) synarcual cartilages is difficult to detect, but it clearly appears remarkably shorter than the posterior (thoracolumbar) synarcual (Fig. 3). It must be pointed out that the concept of thoracolumbar synarcual is still rather poorly defined. This structure, considered a synapomophy of all stingrays, seems to be formed by a true synarcual and, after that, a region of vertebral column in which there are clearly separate vertebrae but their lateral extensions are fused with the degree of fusion getting lesser as they get more distal (C. Underwood pers. obs.). As such, almost the entire vertebral column of †*Lessiniabatis* gen. nov., except for the tail vertebrae, is stiffened by the thoracolumbar synarcual, which is extremely long and extends backward beyond the pelvic girdle (Fig. 4), a condition that is unique in stingrays in which the posterior end of the thoracolumbar synarcual usually lies at mid-length between the pectoral and pelvic girdles^3^. The vertebral column of †*Lessiniabatis* gen. nov. consists of about 120 vertebral centra, including 54 from the first distinguishable centrum to the anterior margin of the puboischiadic bar, and 65–68 from the anterior margin of the puboischiadic bar to the tip of the tail. The vertebral centra are very small, subrectangular in shape and slightly longer than wide. In all the specimens the vertebrae extend to the distal tip of the tail, contrary to the condition in dasyatids, potamotrygonids and pelagic stingrays in which the distal portion of the tail is stiffened by a cartilaginous rod^3^. Ribs are absent.

### Pectoral girdle and fins

**Figure 5:**
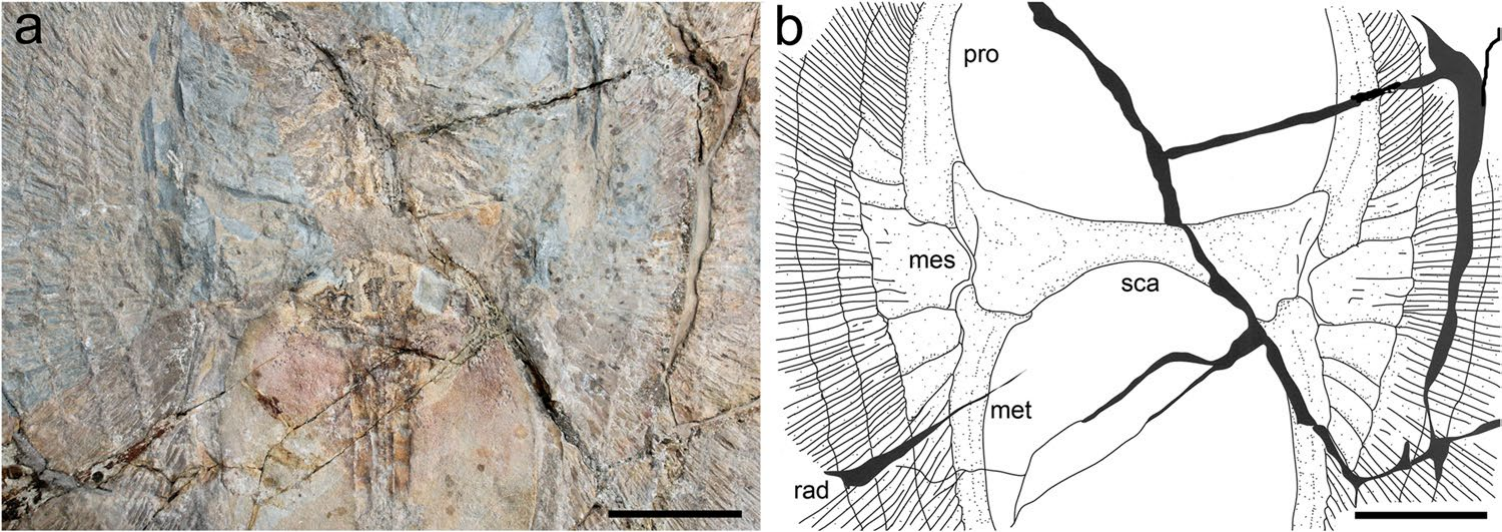
†*Lessiniabatis aenigmatica* gen. et sp. nov. from the Eocene of Bolca Lagerstätte. (**a**) Pectoral girdle region in MNHN F.Bol.566; (**b**) interpretative drawing (gill arches and vertebral column omitted). Abbreviations: mes, mesopterygium; met, metapterygium; pro, propterygium; rad, pectoral radials; sca, scapulocoracoid. Scale bars equal 50 mm.

**Figure 6:**
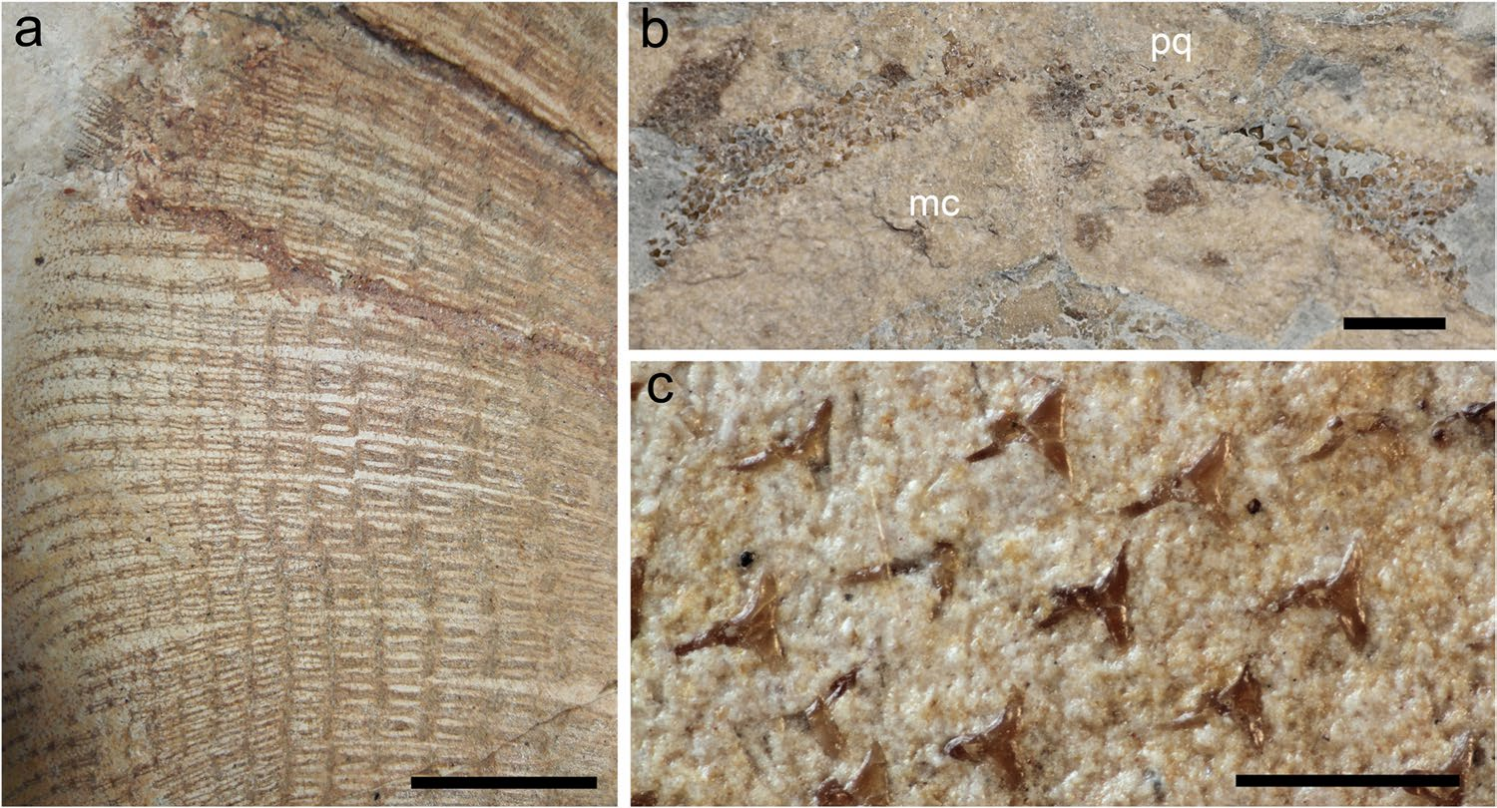
†*Lessiniabatis aenigmatica* gen. et sp. nov. from the Eocene of Bolca Lagerstätte. (**a**) Detail of the pectoral radials in MFSN GP.864 showing the catenated calcification pattern; (**b**) close up of the dentition in MNHN F.Bol.566; (**c**) close up of the dermal denticles in the abdominal region of MFSN GP.864. Scale bars: a = 20 mm; b = 10 mm; c = 1 mm.

The only exposed portion of the scapulocaracoid is the coracoid bar, which is well preserved in MNHN F.Bol.566 as a transverse, straight and robust structure in the middle of the pectoral disc (Fig. 5). Laterally, the coracoid bar articulates with the inner portion of the pterygial skeleton. The propterygium is long and arched, tapering distally and extending to the anterior disc margin. The propterygium is distally segmented; the first small segment lies adjacent to the nasal capsules (Fig. 3). The proximal portion of the propterygium is enlarged, and articulates with the anterior portion of the lateral margin of the scapulocoracoid, and the anterior mesial margin of the mesopterygium (Fig. 5). The mesopterygium appears as a single, large cartilage, whose external margin is more or less straight and fused to the radials. The metapterygium is slightly shorter and more slender than the propterygium. The metapterygium tapers posteriorly, ending near the level of the puboischiadic bar, and is segmented distally. There are about 130–135 pectoral-fin radials of which 59–61 are propterygial, 16–18 mesopterygial, and 54–57 metapterygial. The distribution of the pectoral-fin radials of †*Lessiniabatis* gen. nov. is consistent with that of undulatory swimmers (most of the dasyatoids) in which the metapterygial radials are less than propterygial ones^46^. It is interesting to note that some of the pro-, meso- and metapterygial radials are proximally fused to each other close to the coracoid condyles (Fig. 5). This might be interpreted as an adaptation to strengthen the power of the undulation. Although these structures might be assumed to be fragments of the mesopterygium, those articulating with the proximal portions of the pro- and metapterygia suggest that they most likely represent fused radials, more than fragments of the mesopterygium, which are usually placed between the pro- and metapterygia like in *Gymnura* and some *Myliobatis* species^10,47^. Each radial consists of about 20 segments and bifurcates twice, near its mid-length and near the disc margin. The radials of †*Lessiniabatis* gen. nov. are calcified in a chain-like pattern (Fig. 6a), forming the so-called ‘catenated calcification’ typical for batoids with undulatory swimming mode, including most of the benthic stingrays^48^.

### Pelvic girdle and fins

The pelvic girdle is extremely small, with its width only representing about 8–9% of DW (Fig. 4). It is extremely arched, possibly convergent to the condition seen in *Gymnura*, *Rhinoptera*, and *Mobula*^3^. The long median prepelvic process typical of freshwater potamotrygonids or some pelagic stingrays appears to be absent. Ischial or iliac processes are difficult to recognize. Small, thin pelvic-fin rays lie posteriorly to the puboischiadic bar and articulate with the basypterygia. The pelvic-fin rays are recognizable within the pectoral disc without protruding posteriorly to it. However, their number and structure are difficult to recognize. The presence of claspers is also unclear.

### Dentition

**Figure 7:**
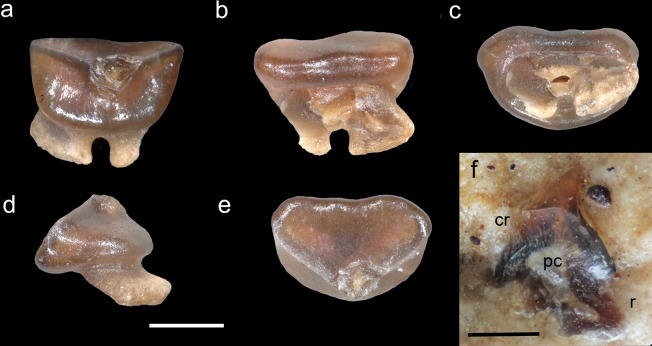
†*Lessiniabatis aenigmatica* gen. et sp. nov. from the Eocene of Bolca Lagerstätte. (**a**–**e**) Isolated (lateral?) tooth from MFSN GP.864 in (**a**) lingual, (**b**) labial, (**c**) basal, (**d**) lateral, and (**e**) labio-occlusal view; (**f**) another (anterior?) tooth from MFSN GP.864 showing its inner vascularization an elongated pulp cavity and vascular tubes of the circumpulpar dentine radiating into crown and root. Abbreviations: cr, tooth crown; pc, pulp cavity; r, root. Scale bars equal 0.5 mm.

The teeth of †*Lessiniabatis* gen. nov. are small, with crown up to 2 mm width and arranged in about 40 rows per jaw in the holotype (Fig. 6b). The dentition appears gradient monognathic heterodont. A single tooth from MFSN GP.864 (Fig. 7a–e) resembles the typical dasyatoid type. The tooth is broad, larger than high, and with a low cusp. The enameloid surface is completely smooth. The lingual surface is broad and slightly convex, whereas the labial surface is smaller, heart-shaped, slightly concave and projects above the root face. The cutting edges are distinct and cutting, sharply separating the labial and lingual crown surfaces. The labial and lingual faces are not steep in lateral profile. The root is bilobed, large and relatively lower than the crown. There is a main central foramen, which opens lingually. Another broken tooth still in place in MFSN GP.864 (Fig. 7f) shows its inner vascularization indicating a holaulacorhizid root type with an elongated pulp cavity in the root area from which the vascular tubes of the circumpulpar orthodentine radiate into the crown.

### Squamation and stings

Although the specimens are mostly preserved in ventral view, as suggested by the exposure of the coracoid bar and dentition, sparse dermal denticles appear to cover the whole body of all individuals (Fig. 6c). Dermal denticles are small (about 500 μm), star-shaped and equally spaced from each other. Since the specimens are possibly exposed in ventral view, the denticles likely show their base, thereby implying that their crown morphology is unknown.

There is no evidence of serrated caudal stings in the tail region of the specimens. Due to the general high quality preservation of the fossils and considering that this structure is easily recognizable in other Bolca stingrays, its absence can be considered a genuine diagnostic character.

#### Phylogenetic analysis

**Figure 8:**
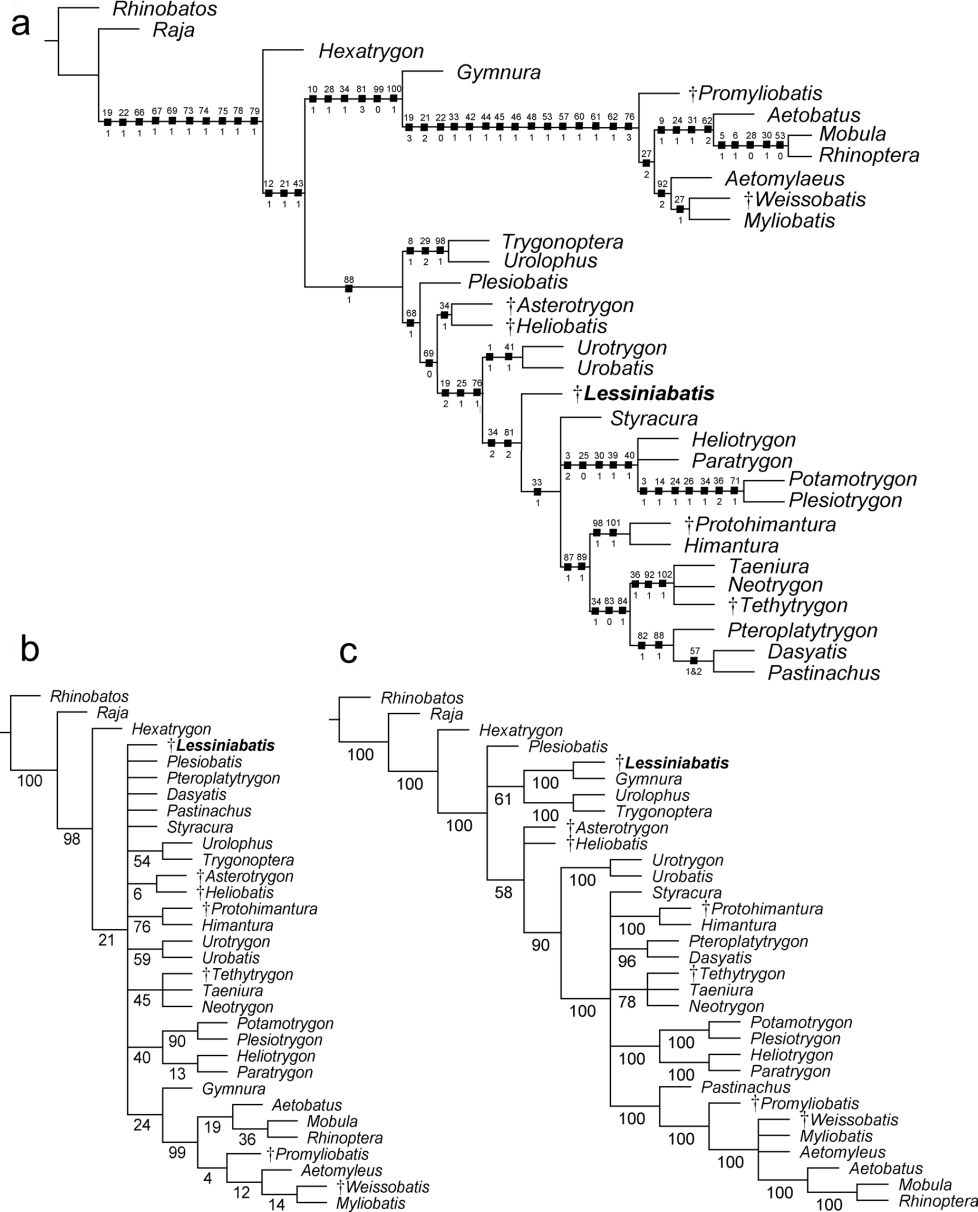
Hypothetical phylogenetic relationships of †*Lessiniabatis aenigmatica* gen. et sp. nov. within the Myliobatiformes. (**a**) The single parsimonious tree retrieved using the branch-and-bound method in TNT. Number character above and state below on each node. (**b**) Bootstrap consensus tree based on the same data matrix. (**c**) 50% Majority rule of 60 trees retrieved using ordered characters. Numbers on nodes are bootstrap values in (**b**,**c**).

The analysis of 102 traits coded for 32 taxa produced a single parsimonious tree with a length of 215 steps, a CI of 0.64, and a RI of 0.80 (Fig. 8a). The tree is consistent with those depicted in recent studies on Bolca stingrays^6,13^. The monophyly of the Myliobatiformes, as recognized by several authors^3,4,49,50^, is confirmed and supported herein (Bremer value 4) by ten characters: basihyal as a single element, but separated from the first hypobranchials (ch. 19[1]); presence of a median projection of the basibranchial medial plate (ch. 22[1]); presence of levator and depressor rostri muscles (ch. 66[1]), serrated tail stings (ch. 67[1]); thorns absent (ch. 69[1]); rostral cartilage vestigial or absent (ch. 73[1]); postorbital process very broad and shelf‐like (ch. 74[1]); jugal arch absent (ch. 75[1]); presence of ball and socket articulation between scapular process and synarcual (ch. 78[1]); presence of a thoracolumbar synarcual (ch. 79[1]).

Like in many other studies primarily based on morphological data^3,4,6,13,45^, the sixgill stingray *Hexatrygon* is inferred to be the sister to all other stingrays, although this contrasts with recent molecular phylogenies, where *Hexatrygon* is recovered as sister to *Gymnura*^16^ or to urolophids^51,52^. The present study detected again a dichotomy of the remaining Myliobatiformes, in which the nature of the two main clades (Myliobatoidea and Dasyatoidea) can be possibly linked to the different calcifications of radial cartilages, body shape, swimming mode and life style^48^. Our analysis detected †*Lessiniabatis* gen. nov. as a genuine member of the benthic stingrays (including Plesiobatidae, Dasyatidae, Potamotrygonidae, Urolophidae, Urotrygonidae and the Eocene stingrays from the Green River Formation, U.S.A.). In particular, †*Lessiniabatis* gen. nov. appears to be more derived than most of the benthic stingrays but is sister to all the potamotrygonids and dasyatids, and sharing with them two characters: the absence of a caudal fin (ch. 34[2]) and the first segment of the propterygium adjacent to nasal capsules (ch. 81[2]). On the contrary, potamotrygonids and dasyatids differ from †*Lessiniabatis* gen. nov. in the presence of a cartilaginous tail rod (ch. 33[1]). Some homoplastic characters can be considered diagnostic for †*Lessiniabatis* gen. nov.: a greatly arched pelvic girdle (ch. 31[1]), absence of tail stings (ch. 67[0]) and thorns (ch. 69[1]). However, the tree loses resolution after bootstrapping and, although some clades are still retrieved (e.g. freshwater potamotrygonids, *Gymnura* plus pelagic stingrays, urolophids, urotrygonids), the relationships of †*Lessiniabatis* gen. nov. within the Myliobatiformes become unclear (Fig. 8b). It is worth to note that using ordered characters †*Lessiniabatis* gen. nov. is retrieved as sister to *Gymnura* (Fig. 8c) sharing with this latter the presence of a greatly arched pelvic girdle (ch. 31[1]) and absence of a caudal fin (ch. 34[2]). Both taxa are placed in a more basal position in the myliobatiform tree and sister to urolophids, in a condition *for Gymnura* more similar to molecular phylogenies^1,4,5^.

## Discussion

**Figure 9:**
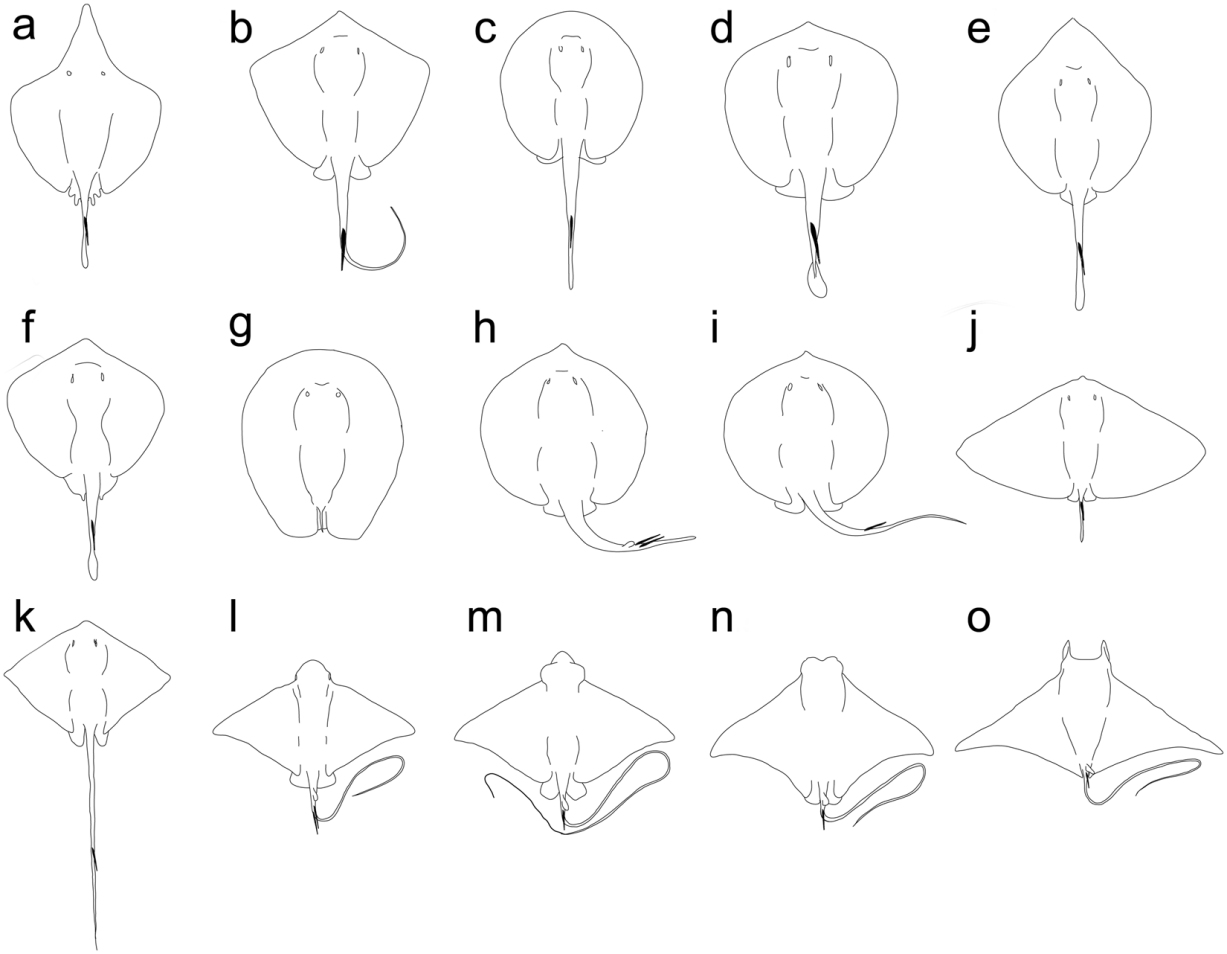
Silhouettes of selected living and fossil taxa as representatives for the modern stingray families and holomorphic fossil taxa. (**a**) *Hexatrygon bickelli* (Hexatrygonidae); (**b**) *Dasyatis marmorata* (Dasyatidae); (**c**) *Potamotrygon tigrina* (Potamotrygonidae); (**d**) *Urobatis halleri* (Urobatidae); (**e**) *Plesiobatis daviesi* (Plesiobatidae); (**f**) *Urolophus kapalensis* (Urolophidae); (**g**) †*Lessiniabatis aenigmatica* gen. et sp. nov.; (**h**) †*Asterotrygon maloneyi*; (**i**) †*Heliobatis radians*; (**j**) *Gymnura altavela* (Gymnuridae); (**k**) †*Promyliobatis gazolai*; (**l**) *Myliobatis hamlyni* (Myliobatidae); (**m**) *Aetobatus laticeps* (Aetobatidae); (**n**) *Rhinoptera bonasus* (Rhinopteridae); (**o**) *Mobula mobular* (Mobulidae). Figures not to scale. Silhouettes are adapted and redrawn from the literature^1,3,13^.

The morphological analysis of these articulated stingray specimens from the Eocene of the Bolca Lagerstätte has revealed the existence of a new stingray taxon whose overall anatomy is unique within the Myliobatiformes (Fig. 9). Despite the bizarre and peculiar anatomy of †*Lessiniabatis* gen. nov., its detailed morphological analysis has revealed the presence of a number of characters that clearly support its inclusion within the order Myliobatiformes, including the absence of a rostral cartilage and the presence of the thoracolumbar synarcual^3,4,8^. The serrated tail sting, considered a synapomorphy of all stingrays, might have been secondary lost in †*Lessiniabatis* gen. nov., a condition that also occurs in some species of *Urogymnus*, *Gymnura*, *Aetomylaeus*, and *Mobula*^1,4^. A combination of several characters argues against the placement of †*Lessiniabatis* gen. nov. within the other stingray lineages. For example, the absence of a caudal fin excludes its assignment to those families characterized by a developed caudal fin (e.g. urolophids and urobatids), whereas the absence of tail folds and a cartilaginous tail rod exclude its assignment to Dasyatidae or Potamotrygonidae.

An external margin of the mesopterygium that is more or less straight and fused to radials rules out the possible relationships between †*Lessiniabatis* gen. nov. and *Gymnura* (undulated, not fused to radials) or the Urolophidae (highly sinuous, fused to radials)^3^. Moreover, the absence of all the shared derived traits characterizing pelagic/benthopelagic stingrays (e.g. crustal calcification of radials, presence of crushing dental plates) supports the exclusion of †*Lessiniabatis* gen. nov. from the group of ‘myliobatid’ stingrays.

The teeth of †*Lessiniabatis* gen. nov. have the typical dasyatoid morphology but their unique combination of features excludes its assignment to any other living and fossil genus. Some Eocene teeth assigned to *Dasyatis* mostly have globular crown with irregular and ornamented surface^53^. In any case, the teeth of these specimens from Bolca are not assignable to *Dasyatis*. The teeth of †*Lessiniabatis* gen. nov. are very different from those of the Eocene gymnurids exhibiting the typical T-shaped crown reduced to cutting crests in occlusal view as in *Gymnura*, or with very steep labial and lingual faces as in †*Jacquhermania*, or characterized by a massive and tall crown typical of †*Ouledia*^53^. The tooth crown of †*Lessiniabatis* does not bear axial lingual folds typical of †*Asterotrygon* also being less high and triangular in outline. †*Lessiniabatis* gen. nov. can be distinguished from †*Arechia* in the absence of a central concavity in the lingual visor that instead characterizes this Eocene dasyatoid genus. The absence of a strong tooth imbrication typical of †*Aturobatis*, as well as the marked enameloid ornamentation, and the very different crown morphologies characteristic of the Eocene dasyatoids †*Coupatezia*, †*Heterotorpedo*, †*Hypolophodon*, †*Merabatis* or †*Meridiania*^53^ allow to exclude any possible alignment of †*Lessiniabatis* gen. nov. to these taxa.

The morphological analysis of †*Lessiniabatis* gen. nov. has revealed the existence of a peculiar body plan unique among Myliobatiformes, which is characterized by a low number of vertebrae posterior to pelvic girdle (65–68), thoracolumbar synarcual extending backward beyond the pelvic girdle, and an extremely short tail, not protruding from the posterior edge of the pectoral disc. The low number of tail vertebrae and the consequent short tail of †*Lessiniabatis* gen. nov. with respect to extant stingrays, might suggest a decreased swimming performance, as the tail and the caudal fin are usually the primary propellers in some batoid clades (e.g. Torpediniformes^54^), in favour of a completely benthic life style, as also suggested by the distribution (mostly propterygial) and calcification pattern (catenated) of the pectoral-fin radials, consistent with that of the benthic undulatory swimmers^46,48^.

Molecular data suggested that stingrays possibly diverged from their sister group, the clade that is today only represented by the genus *Zanobatus*, during the Late Jurassic around 150 million years ago^5,16^. This hypothesis is quite consistent with the fossil record since the oldest stingray (‘*Dasyatis*’ *speetonensis*, whose teeth are morphologically similar to the living *Hexatrygon* than other modern genera) seems to be Hauterivian (Early Cretaceous) in age^11^. A first burst in diversification, with the appearance of pelagic/benthopelagic durophagous stingrays, was in the Late Campanian-Maastrichtian, whereas a second wave of radiation during the late Paleocene-early Eocene saw the appearance and initial radiation of planktivorous taxa^5,16,17^. It is well known that the early Paleogene is also marked by high origination rates of bony and cartilaginous fish lineages, related at least in part to opportunistic ecological niche-filling scenarios in pelagic and benthic realms^18,19,22^ and to the appearance of modern coral reef settings which allow the exploitation of new ecological resources^20,21,23,24^. Moreover, Bolca is also known for the presence of peculiar extinct phenotypes among otherwise familiar bodyplans^27^. From this perspective, the peculiar †*Lessiniabatis* gen. nov. might be considered the evidence of a novel body plan experimented by the Myliobatiformes in the context of the adaptive fish radiation in the aftermath of the end-Cretaceous extinction. However, we cannot exclude the Palaeocene-Eocene Thermal Maximum (PETM) as possible event responsible of the selective pressure that led to the origin of this novel body plan. In any case, the emergence of a new body plan in the Eocene myliobatiforms is particularly intriguing if considered in the context of the coeval extensive adaptive radiation in several bony and cartilaginous fish taxa.

## Methods

This study is based on three nearly complete and articulated specimens housed in the Museum National d’Histoire Naturelle, Paris (MNHN), Museo di Storia Naturale dell’Università di Firenze (MSNFI), and Museo Friulano di Storia Naturale di Udine (MFSN). The specimen MNHN F.Bol.566 has been recently restored^37^. Measurements were taken to the nearest 0.1 mm, and body proportions are detected based on disc width (DW). Osteological and tooth terminology follow primarily the recent literature^3,9,10,53^. The phylogenetic analysis is based on the morphological data set assembled by Marramà *et al*.^13^, which in turn is mainly based on morphology-based phylogenies of stingrays^3,45^ (see Supplementary Information). Character 98 of Marramà *et al*.^13^ (mesio-distally enlarged teeth up to one single tooth row) has been deleted since it is practically coincident with ch. 48 (differentiation of median teeth from lateral teeth). Moreover, since the structure of the mesopterygium in †*Lessiniabatis* gen. nov. appears to be unique among stingrays, a fourth state has been added to ch. 29. The matrix was compiled in Mesquite v.3.03^55^, and the phylogenetic analysis performed with TNT (Tree analysis using New Technology) v.1.5 using the branch‐and‐bound method^56^. All the characters are unordered and given equal weight in the main analysis. Tree length, consistency and retention indices, and Bremer support were subsequently calculated for the single tree retrieved.

## Supplementary information

Supplementary information

## Data Availability

The holotype of †*Lessiniabatis aenigmatica* gen. et sp. nov. (MNHN F.Bol.566 A,B) is housed in the palaeontological collection of the Museum National d’Histoire Naturelle in Paris. The paratypes MSNFI IGF 103555 and MFSN GP.864 are housed in the collections of the Istituto di Geologia del Museo di Storia Naturale dell’Università di Firenze, and Museo Friulano di Storia Naturale in Udine, respectively.
