# Supplementary material for: A bizarre Eocene dasyatoid batomorph (Elasmobranchii, Myliobatiformes) from the Bolca Lagerstätte (Italy) reveals a new, extinct body plan for stingrays
Source: Sci Rep. 2019 Oct 1;9:14087. doi: 10.1038/s41598-019-50544-y (PMC6773687; doi:10.1038/s41598-019-50544-y)
Supplement: Supplementary file 1 — Supplementary information [file 41598_2019_50544_MOESM1_ESM.pdf]

A bizarre Eocene dasyatoid batomorph (Elasmobranchii, Myliobatiformes) from the Bolca Lagerstätte (Italy) reveals a new, extinct body plan for stingrays

Giuseppe Marramà[1]*, Giorgio Carnevale[2], Luca Giusberti[3], Gavin J.P. Naylor[4], Jürgen Kriwet[1]

[1] University of Vienna, Department of Palaeontology, Vienna, 1090, Austria

[2] Università degli Studi di Torino, Dipartimento di Scienze della Terra, Torino, 10125, Italy

[3] Università degli Studi di Padova, Dipartimento di Geoscienze, Padova, 35131, Italy

[4] University of Florida, Florida Museum of Natural History, Gainesville, 32611, Florida, USA

*giuseppe.marrama@univie.ac.at

Supplementary Note 1: Geological setting

Lithological and sedimentological evidence suggest that the specimens object of this study were collected from the fossiliferous layers of the Pesciara site of the Bolca Konservat-Lagerstätte, located in the Monti Lessini (Southern Alps), about 2 km north-east of the village of Bolca, Verona Province, north-eastern Italy. The stratigraphic succession of the Pesciara site is part of the "Calcari Nummulitici", an informal unit of Eocene age widely distributed in north-eastern Italy[1]. The Pesciara site consists of a block of less than 20 m-thick rhythmic alternation of finely laminated micritic limestones including exquisitely preserved fishes, plants and invertebrates, and coarse-grained biocalcarenite/biocalcirudite containing a rich mollusc and alveolinid fauna. The larger benthic foraminiferal and calcareous nannofossil content suggest that the fish-bearing limestone of the Pesciara site belongs to the uppermost part of the SB 11 and lowermost NP 14 (=CNE 6) Zones, corresponding to the late Ypresian, around 49 Ma[1,2]. A recent quantitative palaeoecological and taphonomic study[3] allowed a detailed definition of the palaeoecological and palaeoenvironmental settings of the Bolca palaeobiotopes, confirming that the Pesciara fish assemblage was characterized by a sharp oligarchic structure dominated by zooplanktivorous fishes, whereas taphonomic features confirm that the sediments were deposited in an intraplatform basin in which benthic anoxic conditions and the development of a biofilm acted as promoters of the high-quality preservation of the fossils[1,3].

Supplementary Data 1: Meristic and measurements

Supplementary Table S1. Count and measurements in millimetres (mm) and percentage of disc width (% DW) for the specimens of †*Lessiniabatis aenigmatica* gen. et sp. nov. analysed in this study.

	MNHN F.Bol.566		MSNFI IGF 103555		MFSN GP.864	
**Measurements**	**mm**	**% DW**	**mm**	**% DW**	**mm**	**% DW**
Total length (=disc length)	546.1	117.0	736.5	129.6	468.2	124.8
Disc width	466.8	100.0	568.2	100.0	375.1	100.0
Tail length (from pelvic girdle)	114.6	24.6	190.5	33.5	111.2	29.7
Preoral length	118.5	25.4	165.3	29.1	?	-
Mouth-scapulocoracoid distance	103.5	22.2	152.4	26.8	?	-
Scapulocoracoid width	134.6	28.8	137.5	24.2	96.3	25.7
Pelvic girdle width	38.9	8.3	46.7	8.2	33.5	8.9
Distance from tip of disc to max width disc	262.7	56.3	329.3	58.0	222.5	59.3
Prepelvic distance	400.2	85.7	526.8	92.7	350.6	93.5
Prescapular distance (head length)	224.5	48.1	320.7	56.4	201.6	53.8
**Meristics**						
Propterygial radials	59-61		59-60		60-61	
Mesopterygial radials	16-18		18		?	
Metapterygial radials	54-55		56-57		54	
Total pectoral radials	130-133		133-135		?	
Pelvic radials	?		?		?	
Vertebrae from scapulocoracoid to pelvic girdle	54		?		?	
Vertabrae posterior to pelvic girdle	65		?		68	
Total vertebrae	119		?		?	

Supplementary Data 2: Character list

List of morphological characters used for the phylogenetic analysis, based on Marramà *et al.*[4]. Character 98 of Marramà *et al.*[4] (mesiodistally enlarged teeth up to one single tooth row) has been deleted since it is actually the same as ch. 48 (differentiation of median teeth from lateral teeth).

1. Tubules of subpleural components of hyomandibular lateral line canals: (0) not branched at extremities; (1) extremities dichotomously branched
2. Subpleural components of the hyomandibular lateral line canals: (0) posterior branch extends caudally more or less parallel to longitudinal body axis; (1) posterior branch inflects towards midline to form a lateral hook; (2) posterior branch inflects to continue anteriorly almost parallel to anterior branch, forming a large indentation
3. Suborbital components of infraorbital lateral line canals: (0) projecting posteriorly lateral to mouth; (1) projecting posteriorly lateral to mouth and anteriorly lateral to nasal openings; (2) forming a complex web−like pattern on lateral aspects of the anteroventral disc region
4. Scapular loops formed by scapular components of trunk lateral line canals: (0) absence of loops; (1) presence of scapular loops
5. Anterior process of neurocranium: (0) absent; (1) present
6. Preorbital process: (0) present; (1) absent
7. Preorbital canal for passage of superficial ophthalmic nerve: (0) dorsally located; (1) anteriorly located
8. Foramen for the optic (II) nerve: (0) moderately sized; (1) very enlarged
9. Postorbital process of neurocranium: (0) infraorbital lateral line canal separates postorbital process from small, anterior triangular outgrowth (supraorbital process) of the supraorbital crest; (1) postorbital process with small foramen for passage of infraorbital lateral line canal.
10. Extent of orbital region: (0) orbital region of neurocranium long; (1) shortened orbital region with more anteriorly placed supraorbital and postorbital process

11. Postorbital process: (0) without ventrolateral projection; (1) continuing ventrolaterally to form a cylindrical projection

12. Ventrolateral expansion of nasal capsules: (0) nasal capsules laterally expanded; (1) nasal capsules ventrolaterally expanded

13. Articulation between hyomandibula and Meckel's cartilage: (0) hyomandibulae directly attached to lower jaws; (1) hyomandibulae articulating with lower jaws through strong, stout ligament (hyomandibular−Meckelian ligament) at distal tip

14. Angular cartilages: (0) absence of angular cartilages within hyomandibular−Meckelian ligament; (1) presence of angular cartilages within ligament

15. Secondary hyomandibular cartilages: (0) absent; (1) present

16. Symphysial fusion of upper and lower jaws: (0) antimeres separate at symphysis; (1) both antimeres of jaws symphysially fused.

17. Mandibular width at symphysis: (0) lower jaws slender at symphysis; (1) lower jaws symphysially thickened

18. Lateral projections of lower jaws: (0) absent; (1) present

19. Basihyal cartilage: (0) basihyal laterally elongated, fused to first hypobranchialis; (1) basihyal a single element, but separate from first hypobranchials; (2) basihyal separate from first hypobranchials but fragmented into more than one component; (3) basihyal absent

20. Fusion of ventral pseudohyoid and first ceratobranchial: (0) absent; (1) present

21. Arrangement of posterior ceratobranchials: (0) separate from each other; (1) ankylosis between fourth and fifth ceratobranchials; (2) fourth and fifth ceratobranchials fused to each other.

22. Median projection of the basibranchial medial plate: (0) absent; (1) present

23. Articulation between fifth epi− and ceratobranchial elements to scapulocoracoid: (0) close together; (1) widely separated

24. Lateral stay of synarcual: (0) originates ventral to spinal nerve foramina; (1) originates dorsal to spinal nerve foramina; (2) contacting synarcual both dorsally and ventrally to foramina

25. Fossa on dorsal scapular region: (0) absent; (1) present

26. Contact between pro- and mesopterygium in the pectoral fin: (0) present; (1) absent.

27. Distinct components of the mesopterygium: (0) mesopterygium single element; (1) fragmented; (2) missing altogether.

28. Lateral expansion of radials in pectoral region: (0) absent; (1) present

29. External margin of mesopterygium: (0) more or less straight, not fused to radials; (1) undulated, not fused to radials; (2) highly sinuous, appearing to be fused with articulating radial elements; (3) more or less straight, with fused radials. **Remarks**: since the condition of the mesopterygium in †*Lessiniabatis* gen. nov. appears to be different and unique among stingrays a fourth state (3) has been added.

30. Median prepelvic process: (0) absent or weakly developed; (1) very elongated.

31. Pelvic girdle shape: (0) not arched or only moderately so; (1) greatly arched

32. Dorsal fin: (0) present; (1) absent

33. Cartilaginous rod in tail: (0) absent; (1) present

34. Caudal fin: (0) present; (1) reduced to tail−folds; (2) absent

35. Adductor mandibulae complex: (0) without posteromedial extension; (1) posteromedial extension present

36. Spiracularis muscle: (0) projecting ventrally to insert on either palatoquadrate, Meckel's cartilage, and or hyomandibula; (1) projecting ventrally and posteriorly beyond hyomandibulae and both sets of jaws to insert dorsal to coracomandibularis; (2) projecting ventrally and posteriorly beyond hyomandibulae and both sets of jaws to insert ventral to coracomandibularis

37. Depressor mandibularis muscle: (0) present; (1) absent

38. Coracohyoideus muscle: (0) not connected at midline; (1) connected at midline

39. Urea retention: (0) urea retained in blood; (1) urea excreted in urine

40. Rectal gland: (0) present; (1) reduced

41. Spiracular tentacle: (0) absent; (1) present

42. Cephalic lobes: (0) absent; (1) single and continuous; (2) single with an indentation; (3) paired

43. Nasal curtain: (0) not reaching mouth region; (1) extending posteriorly as far as mouth opening

44. Tooth type in both upper and lower jaws: (0) minute; (1) broad

45. Arrangement of teeth in both upper and lower jaws: (0) arranged in separate diagonal rows or ribbons; (1) horizontal conveyor or pavement−like arrangement

46. Tooth shape: (0) square to rounded; (1) hexagonal, six distinct sides; (2) rectangular with posteriorly deflected lateral margins.

47. Lateral teeth: (0) present; (1) absent

48. Differentiation of median teeth from lateral teeth: (0) median and lateral teeth are similar; (1) median teeth relatively expanded

49. Differentiation among lateral teeth: (0) lateral teeth unexpanded; (1) some lateral teeth expanded

50. Relative amount of curvature in expanded lower teeth: (0) straight and uncurved; (1) moderately curved; (2) strongly curved.

51. Upper tooth curvature: (0) uncurved; (1) curved

52. Direction of tooth curvature: (0) concave; (1) flat/horizontal; (2) convex

53. Tooth association: (0) loosely interlocking; (1) sometimes loosely interlocking or tightly interlocking; (2) tightly interlocking

54. Tooth Interlocking mechanism: (0) overlapping; (1) tongue and groove; (2) no direct contact

55. Shape of interlocking tongue: (0) bulbous; (1) short shelf; (2) long shelf.

56. Crown height: (0) high, the crown height exceeds root depth on unworn teeth; (1) low crown

57. Occlusal surface: (0) cusped; (1) smooth; (2) depressed

58. Crown shape in anterior or posterior view: (0) straight; (1) domed; (2) deep

59. Lateral margins: (0) not pinched; (1) pinched

60. Root type: (0) holaulacorhizous; (1) polyaulacorhizous

61. Number of roots: (0) 2 roots; (1) more than 2 roots.

62. Roots in basal view: (0) triangles; (1) narrow blocks; (2) fine edges.

63. Distance between roots: (0) narrower than root laminae; (1) broad, groove wider than root laminae.

64. Inclination of roots: (0) no inclination; (1) offset and step−like; (2) long and strongly inclined

65. Root groove position: (0) regularly spaced between laminae; (1) irregularly spaced between laminae

66. Levator and depressor rostri muscles: (0) absent; (1) present

67. Serrated tail stings: (0) absent; (1) present.

68. Placoid scales: (0) uniformly present; (1) limited; (2) absent.

69. Thorns: (0) present; (1) absent.

70. Pulp cavities in tooth roots: (0) large; (1) broad and elongated; (2) small; (3) absent

71. (Tooth vascularization: (0) orthodont; (1) osteodont; (2) modified osteodont.

72. (21 of ASC) Infraorbital loop of suborbital and infraorbital canals: (0) absent; (1) present and forming a simple posterolaterally directed loop; (2) present and forming a complex reticular pattern or a number of loops; (3) the loop is directed to the anterior

73. (26 of ASC, modified) Rostral cartilage: (0) complete; (1) vestigial or absent.

74. Postorbital process: (0) narrow; (1) very broad and shelf-like.

75. Jugal arch: (0) present; (1) absent.

76. Basihyal and first hypobranchial: (0) both present and unsegmented; (1) basihyal is segmented; (2) basihyal is absent; (3) basihyal and first hypobranchial cartilages absent

77. Suprascapulae: (0) articulates with vertebral column; (1) fused medially to synarcual (= pectoral arch); (2) fused medially and laterally to synarcual.

78. Ball and socket articulation between scapular process and synarcual: (0) absent; (1) present.

79. Second (thoracolumbar) synarcual: (0) absent; (1) present.

80. Ribs: (0) present; (1) absent.

81. Segmentation of propterygium: (0) posterior to mouth, (1) proximal segment of propterygium of pectoral girdle is between mouth and antorbital cartilage; (2) the first segment is adjacent to the nasal capsule; (3) the first segment is adjacent to anterior margin of antorbital cartilage or anterior to margin of nasal capsule

82. Pseudosiphon: (0) present; (1) absent

83. Dorsal marginal clasper cartilage: (0) lacks medial flange; (1) possesses medial flange

84. Dorsal terminal cartilage: (0) smooth margin; (1) crenate margin

85. Cartilage forming component claw: (0) present; (1) absent; (2) cartilage embedded in integument and is not visible externally; (3) cartilage lines the inner ventral margin of the clasper glans and often forms the component shield

86. Ventral terminal cartilage (accessory terminal 1 cartilage in rajids): (0) simple; (1) free distally and forms component sentinel or is fused with ventral marginal cartilage and forms component projection; (2) folded ventrally along its long axis to form a convex flange

87. Ventral terminal cartilage (accessory terminal 1 cartilage in rajids): (0) attached over length to axial cartilage; (1) free of axial cartilage

88. Spiracularis: 0 = undivided; (1) splits into lateral and medial bundles, with the medial bundle inserting onto the posterior surface of Meckel's cartilage and the lateral bundle inserting onto the dorsal edge of the hyomandibula; (2) extends beyond the hyomandibula and Meckel's cartilage; (3) subdivided proximally and inserts separately onto the palatoquadrate and the hyomandibula.

89. Sexual heterodonty: (0) absent; (1) present.

90. Medial symphyseal processes of the Meckel's cartilage: (0) absent; (1) present.

91. Lateral processes of the palatoquadrate extending far anteriorly: (0) absent; (1) present.

92. Anterior processes of the Meckel's cartilage: (0) absent; (1) present; (2) extending anterior past jaw joint.

93. Lateral oral diastema alt: (0) diastema width greater than occlusal width; (1) occlusal width greater than diastema width.

94. Upper jaw profile: (0) oval in cross-section (most batoids); (1) flat top, convex occlusal surface (myliobatids); (2) strongly flattened (mobulids).

95. Upper jaw mineralization: (0) all surfaces mineralized; (1) lingual face partly unmineralized (mobulids).

96. Lower jaw profile: (0) oval in cross-section; (1) strongly linguolabially expanded.

97. Upper and lower jaw trabeculae: (0) absent; (1) weakly developed; (2) strongly developed.

98. Second transverse keel: (0) absent; (1) present.

99. Calcification pattern of radials: (0) crustal; (1) catenated.

100. Body disc shape: (0) rhombus, quadrangular or oval, with pectoral fins not greatly expanded; (1) wing like, with pectoral fins greatly expanded.

101. Mid-dorsal surface of disc covered by heart-shaped denticles arranged in an antero-posteriorly directed patch having sharply defined outlines: (0) absent; (1) present.

102. File of enlarged ‘caniniform’ teeth in the upper jaw: (0) absent; (1) present.

Supplementary Data 3: Character matrix

	1	2	3	4	5	6	7	8	9	10	11	12	13	14	15	16	17	18	19	20	21	22	23	24	25	26	27	28	29	30
Rhinobatos	0	0	0	0	0	0	0	0	0	0	0	0	0	0	0	0	0	0	0	0	0	0	0	0	0	0	0	0	0	0
Raja	0	0	0	0	0	0	0	0	0	0	0	0	0	0	0	0	0	0	0	0	0	0	0	0	0	0	0	0	0	0
Aetobatus	0	2	0	1	0	0	1	0	1	1	1	1	1	0	1	1	1	1	3	1	2	0	1	1	1	?	2	1	?	0
Aetomylaeus	0	2	0	1	0	0	1	0	[01]	1	1	1	1	0	1	?	1	1	3	1	2	0	1	0	1	0	2	1	0	0
Asterotrygon	?	?	?	?	0	0	?	?	0	0	0	?	1	1	0	0	0	0	1	?	?	1	?	?	0	0	0	0	0	0
Dasyatis	0	1	[01]	1	0	0	0	0	0	0	0	1	1	0	0	0	0	0	2	1	1	1	0	0	1	0	[01]	0	0	0
Gymnura	0	1	0	1	0	0	0	0	0	1	0	1	0	0	0	0	0	0	1	1	1	1	0	0	0	0	1	1	1	0
Heliobatis	?	?	?	?	0	0	?	?	0	0	0	?	1	?	0	0	0	0	?	?	?	?	?	?	0	0	0	0	0	0
Heliotrygon	0	0	2	1	0	0	0	0	0	0	0	1	1	0	0	0	0	0	2	1	1	1	0	0	0	0	0	0	0	1
Hexatrygon	0	0	?	?	0	0	0	0	0	0	0	0	0	0	0	0	0	0	1	?	0	1	0	0	0	0	0	0	0	0
Himantura	0	1	[01]	1	0	0	0	0	0	0	0	1	1	0	0	0	0	0	2	1	1	1	0	0	1	0	0	0	0	0
Lessiniabatis	?	?	?	?	0	?	?	?	?	0	?	?	1	0	?	0	0	0	?	?	?	?	?	?	?	0	0	0	3	0
Mobula	0	2	0	1	1	1	1	0	1	1	1	1	0	0	1	1	0	1	3	1	2	0	1	1	1	?	2	0	?	1
Myliobatis	0	2	0	1	0	0	1	0	0	1	1	1	1	0	1	[01]	1	1	3	1	2	0	1	0	1	0	1	1	0	0
Neotrygon	0	0	0	1	0	0	0	0	0	0	0	1	1	0	0	0	0	0	2	1	1	1	0	0	1	0	0	0	0	0
Paratrygon	0	0	2	1	0	0	0	0	0	0	0	1	1	0	0	0	0	0	2	1	1	1	0	0	0	0	0	0	0	1
Pastinachus	0	1	0	1	0	0	0	0	0	0	0	1	1	0	0	0	0	0	2	1	1	1	0	0	1	0	?	0	0	0
Plesiobatis	0	0	0	1	0	0	0	0	1	0	0	1	1	0	0	0	0	0	1	1	1	0	0	2	0	0	0	0	0	0
Plesiotrygon	0	0	1	1	0	0	0	0	0	0	0	1	1	1	0	0	0	0	2	1	1	1	0	1	0	1	0	0	0	1
Potamotrygon	0	0	1	1	0	0	0	0	0	0	0	1	1	1	0	0	0	0	2	1	1	1	0	1	0	1	0	0	0	1
Promyliobatis	?	?	?	?	0	?	?	?	?	1	?	1	?	?	?	?	?	?	3	1	2	0	?	?	?	?	0	1	0	0
Protohimantura	?	?	?	?	0	0	?	?	?	0	?	?	1	0	0	0	0	0	?	1	1	1	?	?	?	0	0	0	0	?

Pteroplatytrygon	0	1	0	1	0	0	0	0	1	0	0	1	1	0	0	0	0	0	2	1	1	1	0	0	1	0	0	0	0	0
Rhinoptera	0	2	0	1	1	1	1	0	1	1	1	1	1	0	1	1	1	1	3	1	2	0	1	?	1	?	2	0	?	1
Styracura	0	0	0	1	0	0	0	0	0	0	0	1	1	1	0	0	0	0	2	1	1	1	0	0	1	0	0	0	0	0
Taeniura	0	0	0	1	0	0	0	0	0	0	0	1	1	0	0	0	0	0	2	1	1	1	0	0	1	0	0	0	0	0
Tethytrygon	?	?	?	?	0	0	?	?	?	0	?	?	1	0	0	0	0	0	2	1	1	1	?	?	?	0	0	0	0	0
Trygonoptera	0	0	0	1	0	0	0	1	0	0	0	1	1	0	0	0	0	0	1	1	1	1	0	0	1	0	0	0	2	0
Urobatis	1	0	0	1	0	0	0	0	0	0	0	1	1	0	0	0	0	0	2	1	1	1	0	[01]	1	0	0	0	0	0
Urolophus	0	0	0	1	0	0	0	1	1	0	0	1	1	0	1	0	0	0	1	1	1	1	0	0	0	0	0	0	2	0
Urotrygon	1	0	0	1	0	0	0	0	0	0	0	1	1	0	0	0	0	0	3	1	1	1	0	0	1	0	0	0	0	0
Weissobatis	?	?	?	?	0	?	?	?	?	1	?	1	?	?	?	?	?	?	?	?	?	?	?	?	?	?	1	1	0	0

	**31**	**32**	**33**	**34**	**35**	**36**	**37**	**38**	**39**	**40**	**41**	**42**	**43**	**44**	**45**	**46**	**47**	**48**	**49**	**50**	**51**	**52**	**53**	**54**	**55**	**56**	**57**	**58**	**59**	**60**
Rhinobatos	0	0	0	0	0	0	0	0	0	0	0	0	0	0	0	0	0	0	0	?	?	?	0	0	?	0	0	0	0	0
Raja	0	0	0	0	0	0	0	0	0	0	0	0	0	0	0	0	0	0	0	?	?	?	0	0	?	0	0	0	0	0
Aetobatus	1	0	1	2	1	0	1	1	0	0	0	2	1	1	1	2	1	?	?	2	1	2	1	1	2	1	1	0	0	1
Aetomylaeus	0	0	1	2	1	0	1	1	0	0	0	1	1	?	?	1	0	1	0	[01]	[01]	[12]	?	1	1	?	1	?	?	1
Asterotrygon	0	0	0	1	?	?	?	?	?	?	?	0	?	0	0	0	0	0	0	?	?	?	0	0	?	0	0	0	0	0
Dasyatis	0	1	1	1	0	0	0	0	0	0	0	0	1	0	0	0	0	0	0	?	?	?	0	0	?	0	[12]	0	0	0
Gymnura	1	1	0	2	0	0	0	0	0	0	0	0	1	0	0	0	0	0	0	?	?	?	0	0	?	0	0	0	0	0
Heliobatis	0	1	0	1	?	?	?	?	?	?	?	0	?	0	0	0	0	0	0	?	?	?	0	0	?	0	0	0	0	0
Heliotrygon	0	1	1	2	0	0	0	0	1	1	0	0	1	?	?	0	0	0	0	?	?	?	0	?	?	?	?	?	?	?
Hexatrygon	0	1	0	0	0	?	0	?	0	0	0	0	0	0	0	0	0	0	0	?	?	?	0	0	?	0	0	0	0	0
Himantura	0	1	1	2	0	0	0	0	0	0	0	0	1	0	0	0	0	0	0	?	?	?	0	0	?	0	0	0	0	0
Lessiniabatis	1	?	0	2	?	?	?	?	?	?	?	0	?	0	0	0	0	0	0	?	?	?	0	0	?	0	0	0	0	0
Mobula	1	0	1	2	1	0	1	1	0	0	0	3	1	0	1	1	0	[01]	[01]	0	?	1	0	2	?	1	[012]	0	0	1

Myliobatis	0	0	1	2	1	0	1	1	0	0	0	1	1	1	1	1	0	1	0	[01]	[01]	[12]	[01]	1	1	0	1	0	0	1
Neotrygon	0	1	1	1	0	1	0	0	0	0	0	0	1	0	0	0	0	0	0	?	?	?	0	0	?	0	0	0	0	0
Paratrygon	0	1	1	2	0	0	0	0	1	1	0	0	1	0	0	0	0	0	0	?	?	?	0	0	?	0	0	0	0	0
Pastinachus	0	1	1	1	0	0	0	0	0	0	0	0	1	?	?	0	0	0	0	?	0	?	0	0	?	0	[12]	?	?	0
Plesiobatis	0	1	0	0	0	0	0	0	0	0	0	0	1	0	0	0	0	0	0	?	?	?	0	0	?	0	0	0	0	0
Plesiotrygon	0	1	1	1	0	2	0	0	1	1	0	0	1	0	0	0	0	0	0	?	?	?	0	0	?	0	0	0	0	0
Potamotrygon	0	1	1	1	0	2	0	0	1	1	0	0	1	0	0	0	0	0	0	?	?	?	0	0	?	0	0	0	0	0
Promyliobatis	0	?	1	2	?	?	?	?	?	?	?	1	?	1	1	1	0	1	0	1	1	?	1	?	?	?	1	?	?	1
Protohimantura	?	?	?	?	?	?	?	?	?	?	?	?	?	0	0	0	0	0	0	?	?	?	0	0	?	0	0	0	0	0
Pteroplatytrygon	0	1	1	1	0	0	0	0	0	0	0	0	1	0	0	0	0	0	0	?	?	?	0	0	?	0	0	0	0	0
Rhinoptera	1	0	1	2	1	0	1	1	0	0	0	3	1	1	1	1	0	1	1	0	1	2	0	1	0	0	1	0	0	1
Styracura	0	1	1	2	0	2	0	0	0	0	0	0	1	0	0	0	0	0	0	?	?	?	0	0	?	0	0	0	0	0
Taeniura	0	1	1	1	0	1	0	0	0	0	0	0	1	0	0	0	0	0	0	?	?	?	0	0	?	0	0	0	0	0
Tethytrygon	0	1	1	1	?	?	?	?	?	?	?	0	?	0	0	0	0	0	0	?	?	?	0	0	?	0	0	0	0	0
Trygonoptera	0	0	0	0	0	?	0	0	0	0	0	0	1	0	0	0	0	0	0	?	?	?	0	0	?	0	0	0	0	0
Urobatis	0	1	0	0	0	0	0	0	0	0	1	0	1	0	0	0	0	0	0	?	?	?	0	0	?	0	0	0	0	0
Urolophus	0	[01]	0	0	0	0	0	0	0	0	0	0	1	0	0	0	0	0	0	?	?	?	0	0	?	0	0	0	0	0
Urotrygon	0	1	0	0	0	0	0	0	0	0	1	0	1	0	0	0	0	0	0	?	?	?	0	0	?	0	0	0	0	0
Weissobatis	0	0	1	2	?	?	?	?	?	?	?	1	?	1	1	1	0	1	0	1	1	2	1	1	1	0	1	?	0	1

	**61**	**62**	**63**	**64**	**65**	**66**	**67**	**68**	**69**	**70**	**71**	**72**	**73**	**74**	**75**	**76**	**77**	**78**	**79**	**80**	**81**	**82**	**83**	**84**	**85**	**86**	**87**	**88**	**89**	**90**
Rhinobatos	0	0	0	0	?	0	0	0	0	2	0	0	0	0	0	0	0	0	0	0	1	0	0	0	0	0	0	0	0	0
Raja	0	0	0	0	?	0	0	1	0	0	0	0	0	0	0	0	1	0	0	1	0	1	0	0	3	1	1	0	0	0
Aetobatus	1	2	0	2	0	1	1	2	1	3	2	1	1	1	1	3	2	1	1	1	3	0	1	0	2	2	0	?	1	0
Aetomylaeus	1	1	0	?	?	?	[01]	[12]	0	?	2	?	1	?	?	?	2	1	1	1	?	?	?	?	?	?	?	?	0	0

Asterotrygon	0	0	0	0	?	?	1	1	0	?	?	?	1	1	1	0	2	1	1	1	?	?	?	?	?	?	?	?	1	?
Dasyatis	0	0	1	0	?	1	1	[12]	0	3	1	[12]	1	1	1	1	2	1	1	1	3	1	0	1	1	2	1	1	1	1
Gymnura	0	0	0	0	?	1	1	[12]	1	1	0	1	1	1	1	0	2	1	1	1	3	0	1	0	2	2	0	?	0	0
Heliobatis	0	0	0	0	?	?	1	1	0	?	?	?	1	1	1	0	2	1	1	1	?	?	?	?	?	?	?	?	?	?
Heliotrygon	?	?	?	?	?	?	?	?	?	?	?	?	?	?	?	?	?	?	?	?	?	?	?	?	?	?	?	?	0	0
Hexatrygon	0	0	0	0	?	1	1	2	1	1	0	?	1	1	1	0	2	1	1	1	2	?	?	?	?	?	?	?	0	0
Himantura	0	0	0	0	?	1	1	1	0	1	0	2	1	1	1	1	2	1	1	1	3	0	1	0	1	2	1	2	1	1
Lessiniabatis	0	0	0	0	?	?	0	1	1	1	0	?	1	?	?	?	?	?	1	1	2	?	?	?	?	?	?	?	?	?
Mobula	1	?	0	0	[01]	1	1	[12]	1	?	[01]	1	1	1	1	3	2	1	1	1	?	1	?	?	?	?	?	?	[01]	0
Myliobatis	1	1	[01]	0	0	1	1	2	1	3	2	1	1	1	1	3	2	1	1	1	3	0	1	0	2	2	0	0	0	0
Neotrygon	0	0	0	0	?	0	1	[12]	[01]	1	0	1	1	1	0	1	2	1	1	1	2	0	0	1	1	2	1	2	1	0
Paratrygon	0	0	0	0	?	1	1	1	0	?	0	?	1	1	1	1	2	1	1	1	?	0	?	?	?	?	?	2	0	0
Pastinachus	0	0	0	?	?	?	1	1	1	?	2	?	1	?	?	?	2	1	1	1	?	?	?	?	?	?	?	?	0	0
Plesiobatis	0	0	0	0	?	1	1	1	1	?	0	1	1	1	1	0	2	1	1	1	1	0	1	0	?	2	0	1	0	0
Plesiotrygon	0	0	0	0	?	1	1	1	0	?	1	?	1	1	1	1	2	1	1	1	?	0	?	?	?	?	?	2	0	0
Potamotrygon	0	0	0	0	?	1	1	1	0	1	1	3	1	1	1	1	2	1	1	1	2	0	1	0	1	2	0	2	0	0
Promyliobatis	1	1	1	?	0	?	1	2	1	?	?	?	1	?	?	3	?	?	1	1	3	?	?	?	?	?	?	?	?	?
Protohimantura	0	0	0	0	?	?	?	1	?	?	?	?	1	1	?	?	2	?	1	1	3	?	?	?	?	?	?	?	?	?
Pteroplatytrygon	0	0	0	0	?	1	1	1	0	?	1	1	1	1	1	1	2	1	1	1	3	1	0	1	1	2	1	1	1	0
Rhinoptera	1	2	1	1	0	1	1	2	1	3	2	1	1	1	1	3	2	1	1	1	?	0	1	0	2	2	0	3	?	?
Styracura	0	0	0	0	?	1	1	1	0	?	?	1	1	1	1	1	2	1	1	1	2	?	?	?	?	?	?	?	0	0
Taeniura	0	0	0	0	?	1	1	[12]	0	3	1	1	1	1	1	1	2	1	1	1	2	0	0	1	1	2	1	2	1	0
Tethytrygon	0	0	0	0	?	?	1	2	1	?	?	?	1	1		1	2	1	1	1	2	?	?	1	?	?	1	?	1	?
Trygonoptera	0	0	0	0	?	1	1	2	1	?	0	?	1	1	1	?	2	1	1	1	?	0	?	?	?	?	?	?	0	0
Urobatis	0	0	0	0	?	1	1	[12]	0	?	0	1	1	1	1	1	2	1	1	1	1	0	1	0	2	2	0	1	0	0

Urolophus	0	0	0	0	?	1	1	2	1	1	0	1	1	1	1	0	2	1	1	1	1	0	1	0	2	2	0	1	1	0
Urotrygon	0	0	0	0	?	1	1	[12]	0	1	0	1	1	1	1	2	2	1	1	1	1	0	1	0	2	2	0	1	0	0
Weissobatis	1	1	0	0	0	?	1	2	1	?	?	?	1	?	?	?	2	?	1	1	?	?	?	?	?	?	?	?	?	?

	91	92	93	94	95	96	97	98	99	100	101	102
Rhinobatos	0	0	0	0	0	0	0	0	0	0	0	0
Raja	0	0	0	0	0	0	0	0	1	0	0	0
Aetobatus	1	0	0	0	0	1	1	0	0	1	0	0
Aetomylaeus	0	2	0	1	0	1	1	0	0	1	0	0
Asterotrygon	?	?	?	?	?	?	?	0	1	0	0	0
Dasyatis	0	2	0	0	0	0	0	0	1	0	0	0
Gymnura	0	0	1	0	0	0	0	0	0	1	0	0
Heliobatis	?	?	?	?	?	?	?	0	1	0	0	0
Heliotrygon	0	0	0	0	0	0	0	?	?	0	0	0
Hexatrygon	0	0	0	0	0	0	0	0	1	0	0	0
Himantura	0	2	0	0	0	0	0	1	1	0	1	0
Lessiniabatis	?	?	0	?	?	?	?	0	1	0	0	0
Mobula	0	0	1	2	1	1	2	0	0	1	0	0
Myliobatis	1	2	0	1	0	1	1	0	0	1	0	0
Neotrygon	0	1	0	0	0	0	0	0	?	0	0	1
Paratrygon	0	0	1	0	0	0	0	0	1	0	0	0
Pastinachus	0	0	0	0	0	1	0	0	1	0	0	0
Plesiobatis	0	0	0	0	0	0	0	0	0	0	0	0
Plesiotrygon	0	0	0	0	0	0	0	0	1	0	0	0
Potamotrygon	0	0	0	0	0	0	0	0	1	0	0	0

Promyliobatis	?	?	?	?	?	?	?	0	0	1	0	0
Protohimantura	?	?	0	?	0	?	?	1	1	0	1	0
Pteroplatytrygon	0	0	0	0	0	0	0	0	1	0	0	0
Rhinoptera	0	0	?	1	0	1	1	0	0	1	0	0
Styracura	0	?	0	0	0	0	0	0	1	0	0	0
Taeniura	0	1	0	0	0	0	0	0	1	0	0	1
Tethytrygon	?	1	0	?	0	?	?	0	1	0	0	1
Trygonoptera	0	1	0	0	0	0	0	1	1	0	0	0
Urobatis	0	0	0	0	0	0	0	1	1	0	0	0
Urolophus	0	0	0	0	0	0	0	1	1	0	0	0
Urotrygon	0	0	0	0	0	0	0	0	1	0	0	0
Weissobatis	?	?	?	?	?	?	?	0	0	1	0	0

Supplementary References

1. Papazzoni, C. A. & Trevisani, E. Facies analysis, palaeoenvironmental reconstruction, and biostratigraphy of the “Pesciara di Bolca” (Verona, northern Italy): An early Eocene Fossil-Lagerstätte. *Palaeogeogr. Palaeoclimatol. Palaeoecol*. **242**, 21-35 (2006).
2. Papazzoni, C. A., Fornaciari, E., Giusberti, L., Vescogni, A. & Fornaciari, B. Integrating Shallow Benthic and Calcareous Nannofossil Zones: the lower Eocene of the Monte Postale section (northern Italy). *Palaios* **32(1)**, 6-17 (2017).
3. Marramà, G., Bannikov, A. F., Tyler, J. C., Zorzin, R. & Carnevale, G. Controlled excavations in the Pesciara and Monte Postale sites provide new insights about the paleoecology and taphonomy of the fish assemblages of the Eocene Bolca Konservat-Lagerstätte, Italy. *Palaeogeogr. Palaeoclimatol. Palaeoecol*. 454, 228-245 (2016).
4. Marramà, G., Carnevale, G., Naylor, G. & Kriwet J. Mosaic of plesiomorphic and derived characters in an Eocene myliobatiform batomorph (Chondrichthyes, Elasmobranchii) from Italy defines a new, basal body plan in pelagic stingrays. *Zool. Lett.* **5**, 13 (2019).
